# Adipose tissue deficiency of hormone-sensitive lipase causes fatty liver in mice

**DOI:** 10.1371/journal.pgen.1007110

**Published:** 2017-12-12

**Authors:** Bo Xia, Guo He Cai, Hao Yang, Shu Pei Wang, Grant A. Mitchell, Jiang Wei Wu

**Affiliations:** 1 College of Animal Science and Technology, Northwest A&F University, Yangling, Shaanxi, China; 2 Division of Medical Genetics, Department of Pediatrics, Université de Montréal and CHU Sainte-Justine, Montréal, QC, Canada; Stanford University School of Medicine, UNITED STATES

## Abstract

Fatty liver is a major health problem worldwide. People with hereditary deficiency of hormone-sensitive lipase (HSL) are reported to develop fatty liver. In this study, systemic and tissue-specific HSL-deficient mice were used as models to explore the underlying mechanism of this association. We found that systemic HSL deficient mice developed fatty liver in an age-dependent fashion between 3 and 8 months of age. To further explore the mechanism of fatty liver in HSL deficiency, liver-specific HSL knockout mice were created. Surprisingly, liver HSL deficiency did not influence liver fat content, suggesting that fatty liver in HSL deficiency is not liver autonomous. Given the importance of adipose tissue in systemic triglyceride metabolism, we created adipose-specific HSL knockout mice and found that adipose HSL deficiency, to a similar extent as systemic HSL deficiency, causes age-dependent fatty liver in mice. Mechanistic study revealed that deficiency of HSL in adipose tissue caused inflammatory macrophage infiltrates, progressive lipodystrophy, abnormal adipokine secretion and systemic insulin resistance. These changes in adipose tissue were associated with a constellation of changes in liver: low levels of fatty acid oxidation, of very low density lipoprotein secretion and of triglyceride hydrolase activity, each favoring the development of hepatic steatosis. In conclusion, HSL-deficient mice revealed a complex interorgan interaction between adipose tissue and liver: the role of HSL in the liver is minimal but adipose tissue deficiency of HSL can cause age-dependent hepatic steatosis. Adipose tissue is a potential target for treating the hepatic steatosis of HSL deficiency.

## Author summary

Fatty liver is a major complication of obesity and of type 2 diabetes mellitus. It carries a high risk of cirrhosis and liver cancer. In fatty liver, triglycerides accumulate to high levels in the cytoplasm of hepatocytes. Triglycerides are degraded by lipolysis, which has been most studied in fat cells where its three steps are catalyzed by different enzymes. The second step, hydrolysis of diglyceride to a monoglyceride, can be mediated by hormone-sensitive lipase (HSL). Patients with genetic deficiency of HSL have fatty liver. In this study, we found that systemic HSL deficient mice developed fatty liver with aging. To study the mechanism of steatosis, we made liver-specific HSL-deficient mice. Surprisingly, these mice had normal liver fat content. We then studied mice with HSL deficiency in adipose tissue. Adipose HSL-deficient mice developed hepatic steatosis to a similar extent as mice with systemic HSL deficiency, showing that adipose HSL deficiency is sufficient to cause fatty liver. Furthermore, like reported HSL-deficient humans, mice with adipose HSL deficiency had systemic insulin resistance, reduced fat mass and inflammation in fat tissue. Each of these is known to promote hepatic steatosis. Livers of adipose HSL-deficient mice showed low levels of hepatic fatty acid (FA) oxidation, of very low density lipoprotein (VLDL) secretion and of triglycerides (TG) hydrolase activity, each of which could contribute to fat accumulation in liver. Tissue-selective genetic alterations may help in identifying and understanding the tissues responsible for complex metabolic phenotypes like fatty liver. Our data suggest that at least in mice, strategies for treatment of fatty liver related to HSL deficiency should concentrate on adipose tissue.

## Introduction

Disorders of lipid accumulation such as obesity and fatty liver (hepatic steatosis) are among the greatest risk factors for health in developed countries [1–4]. Hepatic steatosis is linked to the development of liver fibrosis, cirrhosis and cancer [5, 6] and is rapidly increasing in prevalence [7, 8]. Increasing interest centers on the biology of triglyceride (TG)-containing cytoplasmic lipid droplets, TG synthesis and TG degradation (lipolysis). In adipose tissue, hormone-sensitive lipase (HSL), a cytoplasmic lipase encoded by the *LIPE* gene, is important for lipolysis. After the initial cleavage of a TG to a diacylglycerol (DG) plus a fatty acid (FA), performed by adipose triglyceride lipase (ATGL) [9], HSL-mediated hydrolysis of DG to a monoacylglycerol (MG) plus a FA [10]. MG is cleaved in turn by a MG hydrolase to release glycerol plus a FA. In humans and mice, systemic ATGL deficiency causes hepatic steatosis [11, 12]. By using liver-specific ATGL deficient mouse models, we and others further showed that ATGL deficiency in liver causes marked hepatic steatosis in mice, suggesting that the underlying mechanism of ATGL-related hepatic steatosis is liver autonomous [13, 14].

As in ATGL deficiency, the small number of individuals with genetic deficiency of HSL reported so far also show liver steatosis in middle age [15]. The underlying mechanism of HSL-related hepatic steatosis is still elusive. HSL-deficient mice have been described and, like HSL-deficient humans, show protection from obesity, a low capacity to increase lipolysis following beta-adrenergic stimulation and higher levels of diglycerides in adipose tissue [10, 16, 17]. Paradoxically, some HSL-deficient mouse strains have been reported to develop hepatic steatosis [18, 19] but other reports mention low liver fat content in HSL-deficient mice [10, 20, 21].

To explore the potential mechanism of HSL deficiency-related hepatic steatosis, we studied the effect of HSL deficiency on liver fat content in different mouse models. The result show that hepatic steatosis occurs with aging in HSL-deficient mice. Using three models of HSL deficiency (systemic, hepatic and adipose) we show that, surprisingly, unlike ATGL, liver fat levels are unrelated to liver HSL but that adipose HSL deficiency alone is sufficient to produce a similar level of hepatic steatosis as in systemic HSL deficiency.

## Results

### HSLSKO mice have age-dependent hepatic steatosis

**Fig 1:**
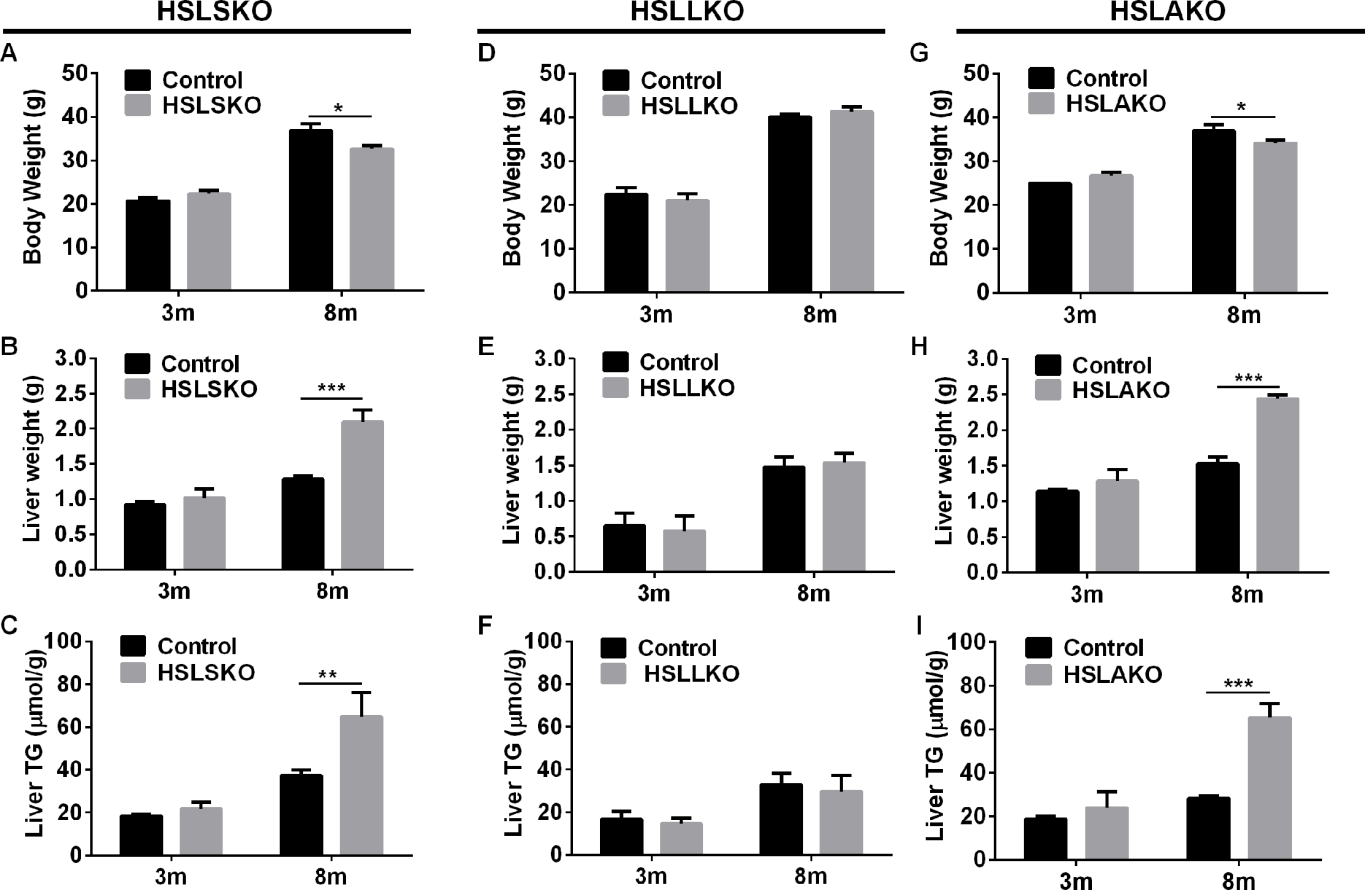
Age-dependent hepatic steatosis occurs in HSLSKO and HSLAKO mice but not in HSLLKO mice. 5-hour-fasted 3-month-old and 8-month-old mice were used (n = 6). A. HSLSKO body weight; B. HSLSKO liver weight; C. HSLSKO liver TG content; D. HSLLKO body weight; E. HSLLKO liver weight; F. HSLLKO liver TG content; G. HSLAKO body weight; H. HSLAKO liver weight; I. HSLAKO liver TG content. HSLSKO, systemic HSL knockout mice; HSLLKO, liver HSL knockout mice; HSLAKO, adipose HSL knockout mice. *, p < 0.05; **, p<0.01; ***, p<0.001 for all figures.

Comparison of reports of liver fat content of HSL-deficient mice revealed that studies reporting low liver TG content [10, 20, 21] were performed in mice before 4 months of age, whereas all those reporting hepatic steatosis [18, 19] were in older mice. To test the hypothesis that systemic HSL knockout (HSLSKO) mice develop hepatic steatosis with aging, two groups of mice were studied, aged 3 and 8 months. Three-month-old HSLSKO mice had similar body weight (Fig 1A), liver weight (Fig 1B) and liver fat content (Fig 1C) to controls. However, at 8 months of age, although HSLSKO mice were lean (Fig 1A), their liver mass (Fig 1B) and TG content (Fig 1C) were greater than those of controls. Therefore, available data show the age-dependent development of hepatic steatosis in HSLSKO mice.

### Liver HSL does not contribute to hepatic steatosis

To investigate the mechanism of hepatic steatosis in HSLSKO mice, we hypothesized that HSL, which is essential for normal lipolysis in adipose tissue, might also be important for degradation of acylglycerols in liver and thus directly influence liver fat content. To test this, liver-specific HSL knockout (HSLLKO) mice were created. Deficiency of HSL in liver was demonstrated by the absence of detectable HSL protein S1A Fig, and very low HSL mRNA S1B Fig in liver. Surprisingly, in contrast to HSLSKO mice, HSLLKO mice were similar to normal controls in body weight (Fig 1D), liver weight (Fig 1E) and liver TG content (Fig 1F). These results proved that hepatic HSL does not contribute to fatty liver in HSL deficiency, suggesting that the mechanism of hepatic steatosis in HSL deficiency depends upon organs other than liver.

### HSL deficiency in adipose tissue leads to hepatic steatosis

**Fig 2:**
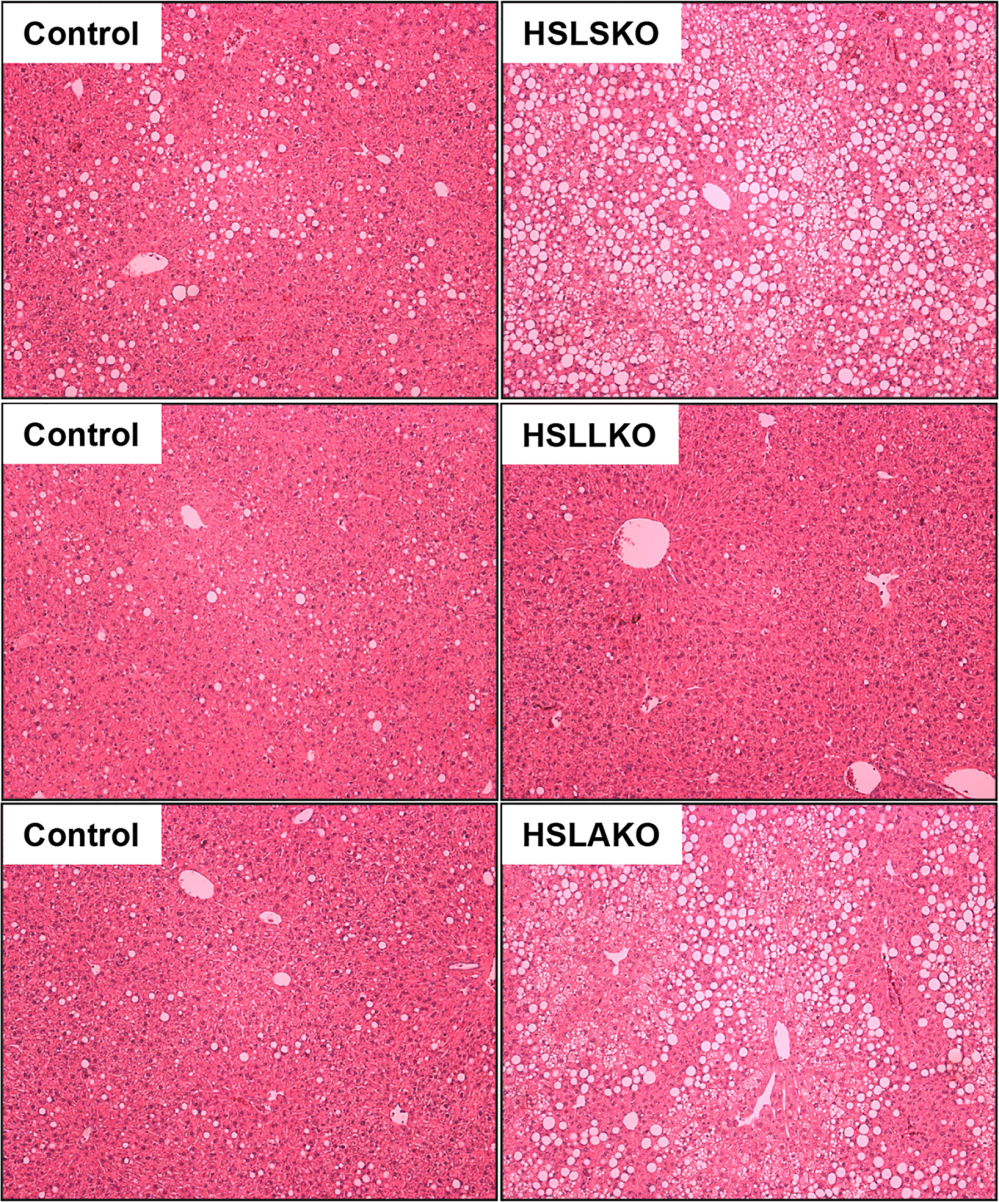
Histological confirmation of hepatic steatosis in HSLSKO and HSLAKO livers, but not in HSLLKO liver. 8-month-old mice were fasted for 5 hours. Representative H&E sections of liver are shown from mice of each genotype.

Because adipose tissue is a major regulator of TG storage and of FA release, we hypothesized that HSL deficiency in adipose tissue might cause systemic metabolic changes leading to hepatic steatosis. To test this, mice with adipose HSL deficiency (HSLAKO) were created as described [22]. Compared to normal controls, at 3 months of age, HSLAKO mice had similar body weight (Fig 1G), liver weight (Fig 1H) and liver TG content (Fig 1I). However, at 8 months of age, HSLAKO mice showed lower body weight (Fig 1G), but higher liver mass (Fig 1H) and higher liver TG content (Fig 1I) than controls. The severity of the steatosis of HSLAKO mice was similar to that observed in HSLSKO mice (Fig 1C and 1I). Liver histology of 8-month-old mice confirmed these findings, showing hepatic steatosis in HSLSKO and HSLAKO mice, but not in HSLLKO mice (Fig 2). Therefore, HSL deficiency in adipose tissue alone is sufficient to cause the age-dependent hepatic steatosis observed in systemic HSL deficiency.

### Adipose tissue HSL deficiency causes lipodystrophy

**Fig 3:**
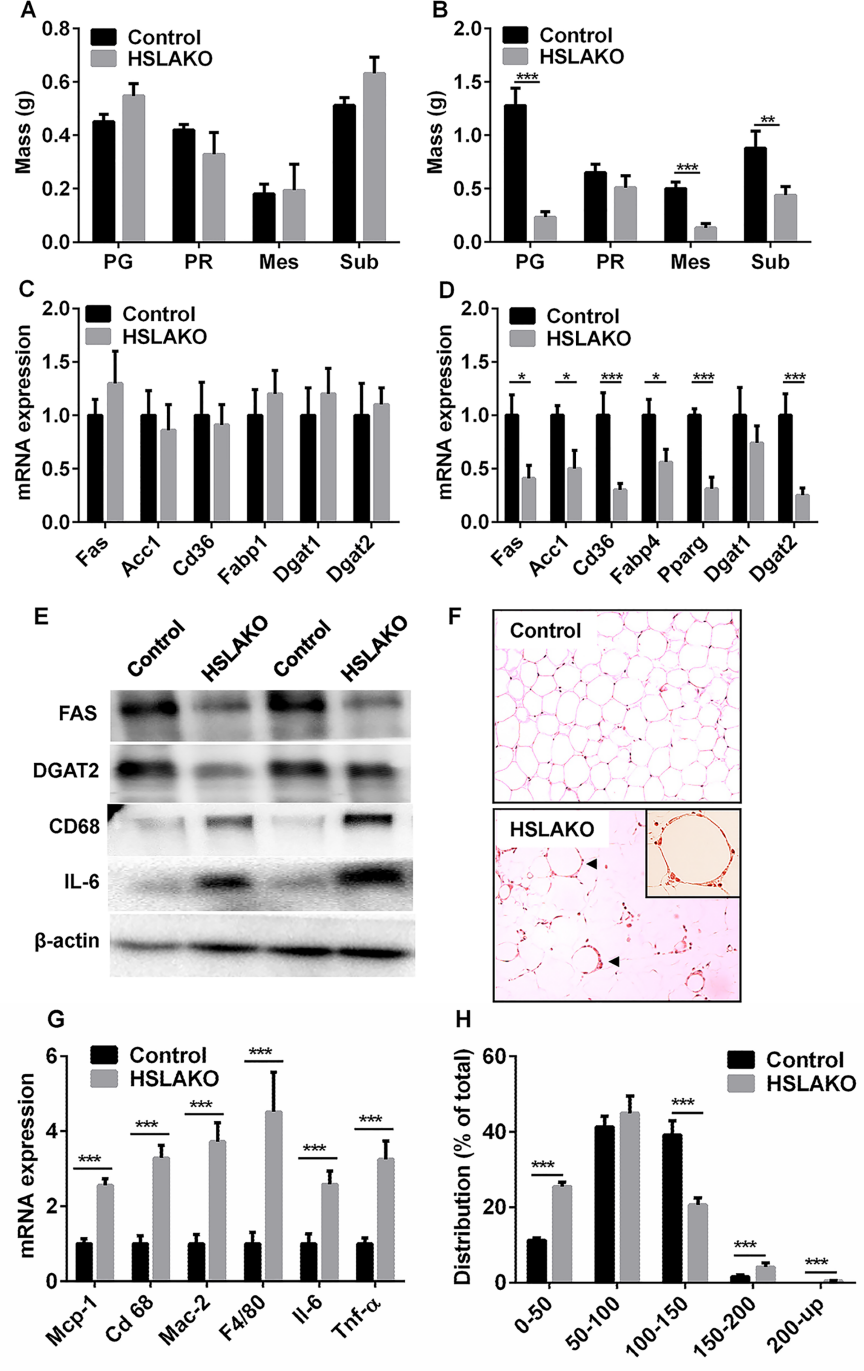
Lipodystrophy and macrophage infiltration of white adipose tissue of HSLAKO mice. A. masses of different fat depots at 3 months. B. masses of different fat depots at 8 months. Mice were fasted for 5 hours (n = 6). PG, perigonadal; PR, perirenal; Mes, mesenteric; Sub, subcutaneous fat. C. 3-month-old mice and D. 8-month-old mice adipose expression of transcripts related to TG metabolism. *Fas*, fatty acid synthase; *Acc1*, acetyl-CoA carboxylase 1; *Cd36*, cluster of differentiation 36, (a transporter for fatty acids); *Fabp4*, fatty acid binding protein 4; *Ppar-γ*, peroxisome proliferator-activated receptor gamma; *Dgat1*, diacylglycerol O-acyltransferase 1; *Dgat2*, diacylglycerol O-acyltransferase 2. Mice fasted for 5 hours were used (n = 6). E. Protein expression in adipose tissue. Western blots of the indicated proteins, 8-month-old mice adipose tissue. F. H&E staining of white adipose tissue, showing the high prevalence of crown-like structures (arrows), X100; Inset, enlarged image of a crown-like structure, > 200; G. markers of macrophage and of inflammation in adipose tissue (n = 6); H. distribution of adipocyte diameter. 8-month-old mice fasted for 5 hours were used.

Compared to matched controls, 3-month-old HSLAKO mice had similar body weight to controls (Fig 1G), but 8-month-old HSLAKO mice had lower body weight (Fig 1G). Further measurements showed that HSLAKO mice have similar fat mass as controls at 3 months (Fig 3A), but lower mass at 8 months (Fig 3B). Consistent with this, markers of lipogenesis and of TG synthesis in adipose tissue, including *Fas*, *Acc1*, *Cd36*, *Fabp4*, *Ppar-γ* and *Dgat2*, were similar between HSLAKO mice and the corresponding controls at 3 months (Fig 3C), but significantly lower in 8-month-old HSLAKO adipose tissue than in the corresponding controls (Fig 3D). To further explore lipogenesis and TG synthesis in 8-month-old HSLAKO adipose tissue, we studied the expression of key proteins of lipogenesis (FAS) and of TG synthesis (DGAT2). As seen in the corresponding mRNAs, FAS and DGAT2 protein levels were lower than those of controls (Fig 3E). Histologically, adipose tissue of HSLAKO mice showed macrophage infiltration (Fig 3F) and increased levels of macrophage and inflammatory markers (Fig 3E and 3G). HSLAKO white adipose tissue (WAT) had heterogeneity of cell size (Fig 3H), with a bimodal distribution in which small adipocytes (≤50 μm) and large adipocytes (≥150 μm) are each significantly more prevalent than in controls (Fig 3H). Together, these results show that HSL deficiency in adipose tissue causes age-related lipodystrophy, with decreased fat mass and inflammation in adipose tissue.

### HSL deficiency in adipose tissue causes systemic changes in energy homeostasis

**Fig 4:**
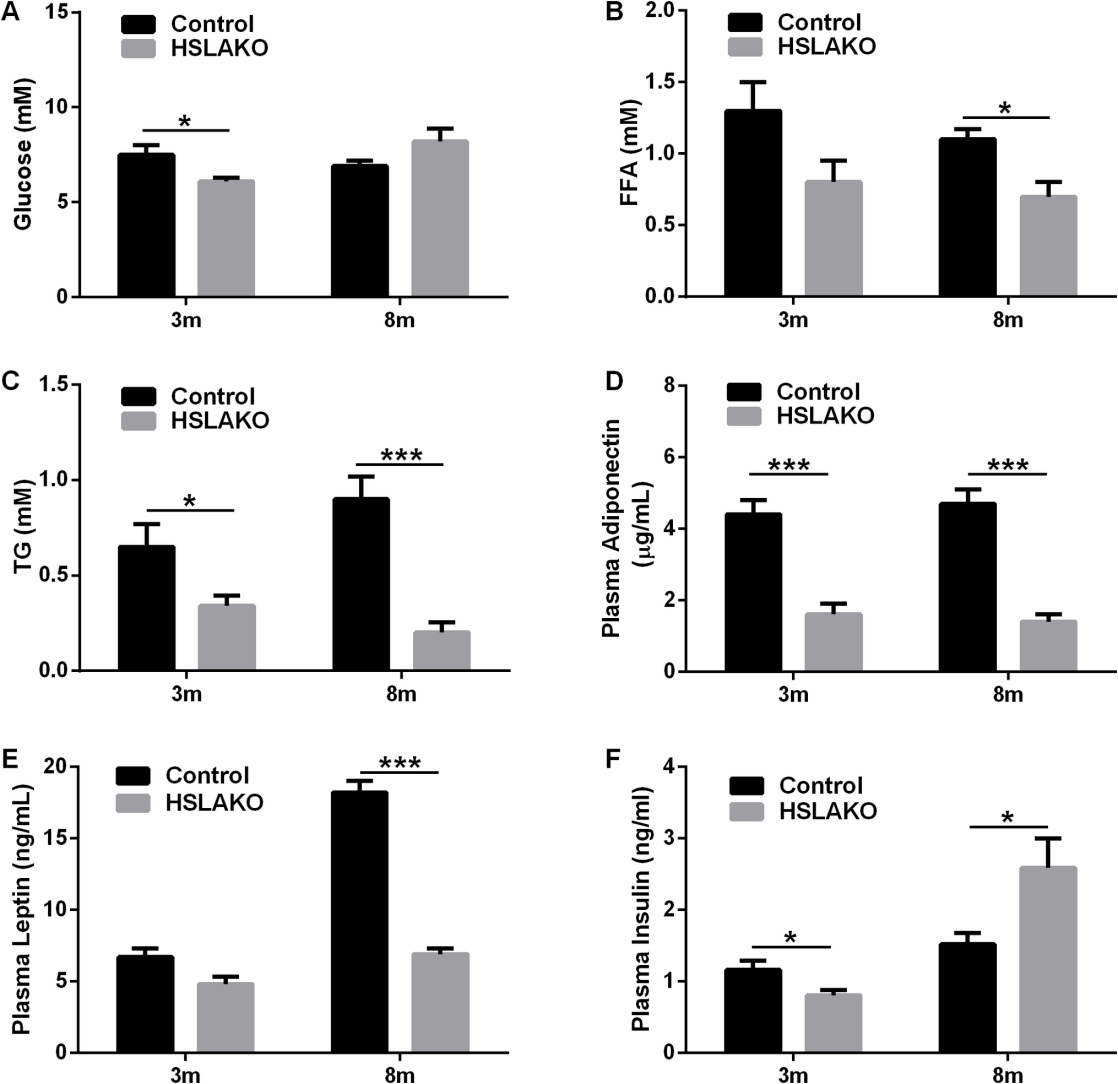
Plasma levels of energy-related metabolites and hormones in HSLAKO mice. 14-hour overnight fasted mice were used for the following measurements: A. Glucose level; B. FFA level and C. TG level. 5-hour fasted mice were used for the following: D. Adiponectin. E. Leptin and F. Insulin. n = 6.

Plasma metabolites related to energy metabolism were measured in 14-hour overnight fasted mice. The result showed that plasma glucose was lower in 3-month-old HSLAKO mice than in corresponding controls (Fig 4A). No difference in plasma glucose was observed in 8-month-old mice (Fig 4A). Plasma FA level was not significantly different from controls values in 3-month-old HSLAKO mice (Fig 4B), but was significantly lower in 8-month-old HSLAKO mice (Fig 4B). Compared to normal controls, HSLAKO mice showed lower levels of plasma TG (Fig 4C) and of plasma adiponectin (Fig 4D) both at 3 and at 8 months of age. HSLAKO mice failed to show the age-related increase in leptin levels seen in normal controls (Fig 4E). Interestingly, we found that 3-month-old HSLAKO mice had lower levels of insulin than controls, but 8-month-old HSLAKO mice had higher levels (Fig 4F), suggesting an age-related development of insulin resistance.

**Fig 5:**
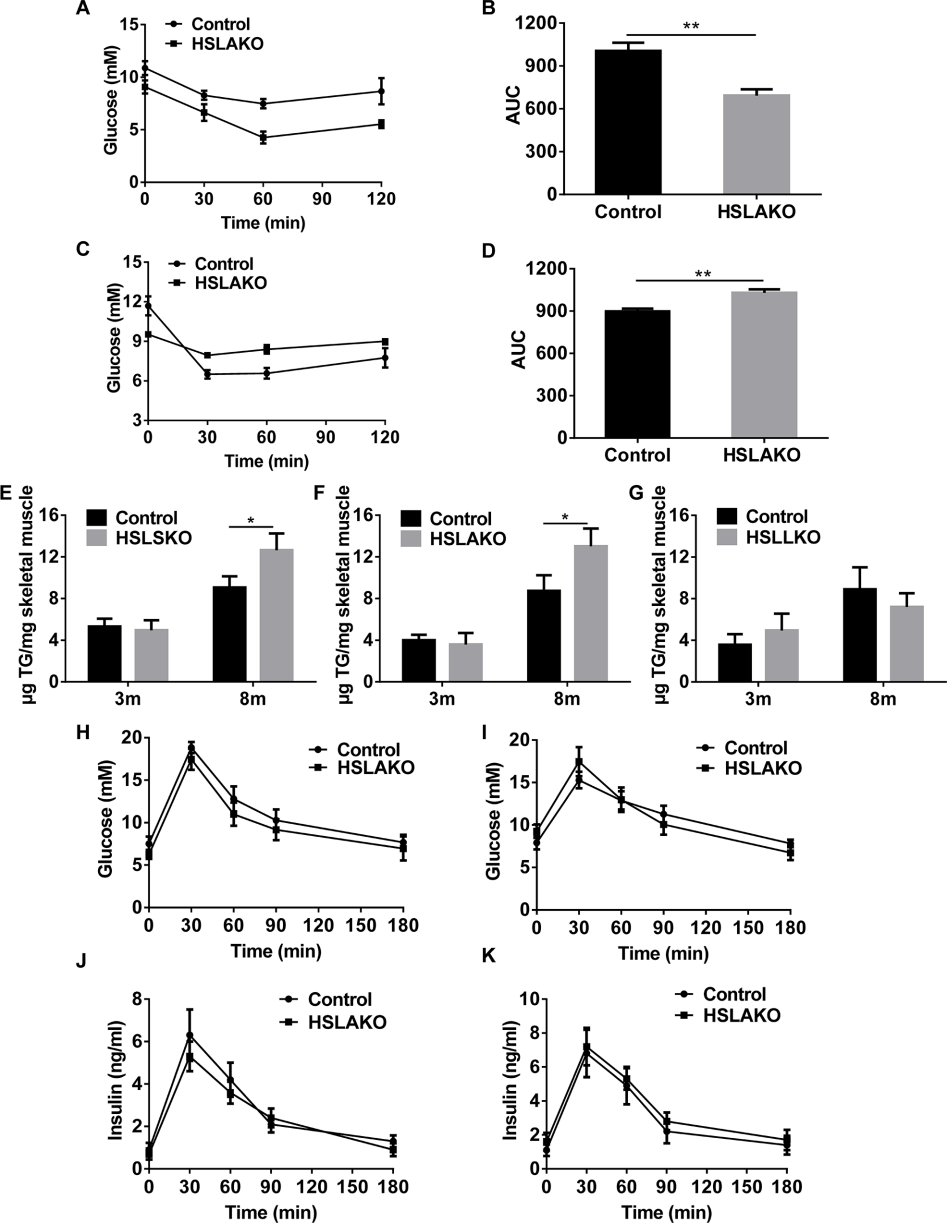
Insulin sensitivity and glucose tolerance in HSLAKO mice. A. ITT for 3-month-old mice. B. Area under the curve (AUC) for ITT at 3 months. C. ITT for 8-month-old mice. D. AUC for ITT at 8 months. E, F and G. skeletal muscle TG content in HSLSKO, HSLAKO and HSLLKO mice, respectively. H, I. Glucose levels during the GTT in 3- and 8-month-old mice. J, K. Insulin levels during the GTT at 3 and 8 months, respectively.

To further test insulin sensitivity, we performed insulin tolerance tests in HSLAKO and control mice. Compared to normal controls, HSLAKO mice showed improved insulin sensitivity at 3 months (Fig 5A–5B) but were insulin resistant at 8 months of age (Fig 5C–5D), demonstrating that systemic insulin resistance develops with age in HSLAKO mice. Iinsulin sensitivity is driven in large part by insulin-stimulated muscle glucose uptake, and this is suppressed by muscle fat content. Therefore, muscle fat content was measured in the HSLSKO, HSLLKO, and HSLAKO mice. As with liver TG content, HSLSKO and HSLAKO mice showed similar levels of skeletal muscle fat content to their corresponding controls at 3m, but higher levels at 8m (Fig 5E–5F). This difference was not seen in 8 month old HSLLKO mice (Fig 5G). Glucose tolerance was similar in HSLAKO mice and normal controls (Fig 5H–5K).

In summary, primary adipose deficiency of HSL in mice results in atrophy and inflammation of adipose tissue and systemic insulin resistance. Each of these has been reported to promote hepatic steatosis [23–25]. We next studied the pathways of TG and FA disposal in liver, including FA oxidation, TG export in VLDL and TG hydrolysis to see whether one or more might be affected by adipose HSL deficiency.

### FA oxidation is low in HSLAKO liver

**Fig 6:**
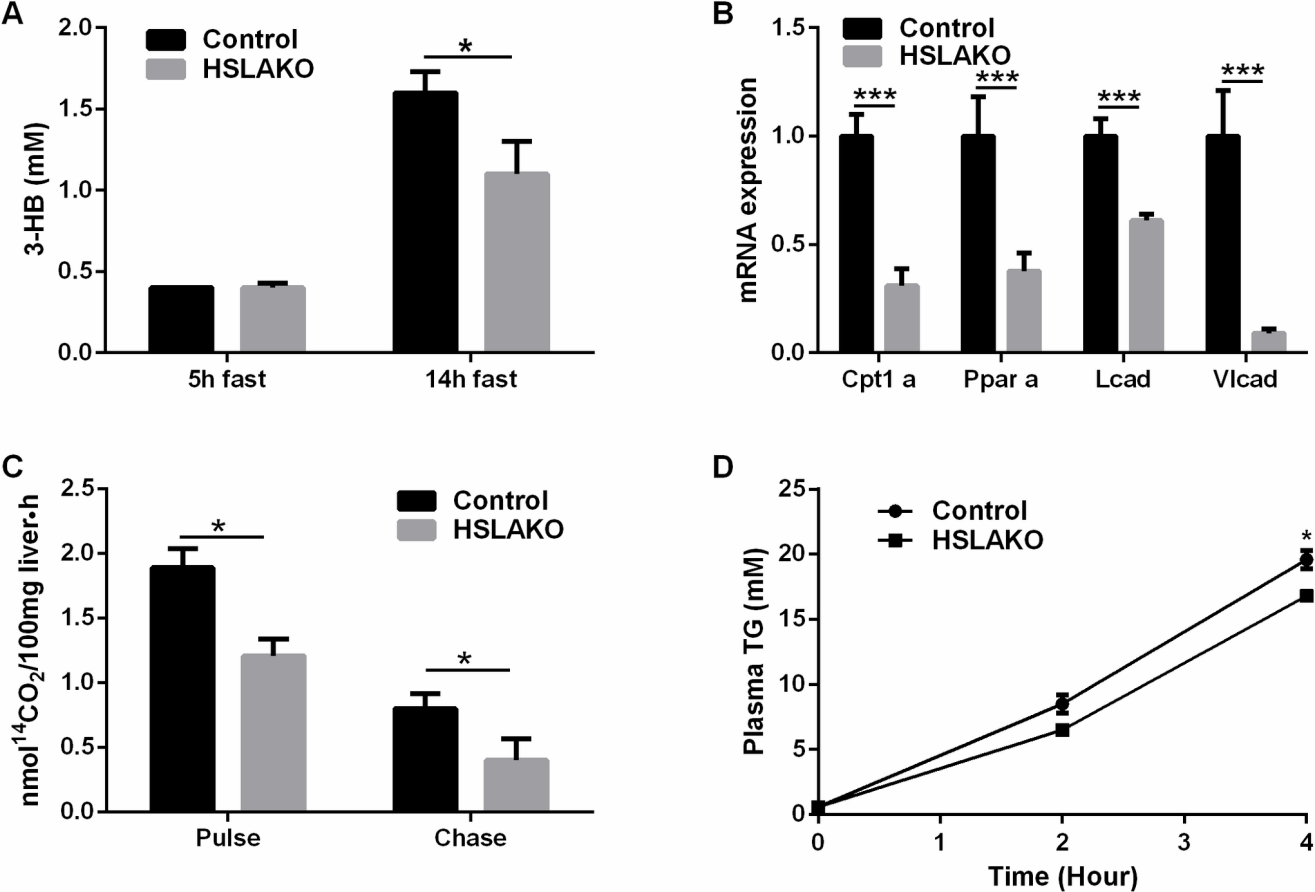
Low levels of ketogenesis, fatty acid oxidation and VLDL production in HSLAKO livers. A. plasma 3-hydroxybutyrate (3-HB). B. transcripts related to β-oxidation in liver. C. β-oxidation in liver slices, measured as production of CO_2_ from FA substrates. 8-month-old mice were used (n = 6). D. plasma TG levels following injection of the LPL inhibitor, P407.

Plasma 3-hydroxybutyrate (3-HB) levels provide one indication of liver FA oxidation and ketone body production. After a 5-hour fast, 3-HB levels were similar in HSLAKO and control mice, but after a 14-hour fast, 3-HB levels were significantly lower in HSLAKO mice than controls (Fig 6A), consistent with lower hepatic FA oxidation and ketogenesis. Also, low expression of genes related to FA oxidation, including *Cpt1a*, *Pparα*, *Lcad* and *Vlcad*, was observed in HSLAKO liver (Fig 6B). Finally, hepatic FA oxidation was measured directly in liver slices using 1-^14^C palmitic acid as substrate. HSLAKO livers had a lower rate of FA oxidation than control livers (Fig 6C). Together, these results suggest that HSLAKO mice had lower hepatic FA oxidation than controls. This could contribute to hepatic steatosis.

### Hepatic VLDL production is low in HSLAKO mice

Reduction of hepatic release of TG, measured as VLDL secretion, could also contribute to hepatic steatosis [26]. To test whether adipose HSL deficiency affects hepatic VLDL production, we measured the increase of plasma TG level 4 hours after intraperitoneal injection of poloxamer 407 (P407), an inhibitor of lipoprotein lipase. Compared to normal controls, HSLAKO mice showed lower post-P407 plasma TG levels at 4h after injection (Fig 6D), indicating lower production of VLDL. These results demonstrate lower production of VLDL in HSLAKO mice than in controls. This could potentially contribute to hepatic steatosis in HSLAKO mice.

### Hepatic TG hydrolase activity and lipolytic gene expression are low in HSLAKO mice

**Fig 7:**
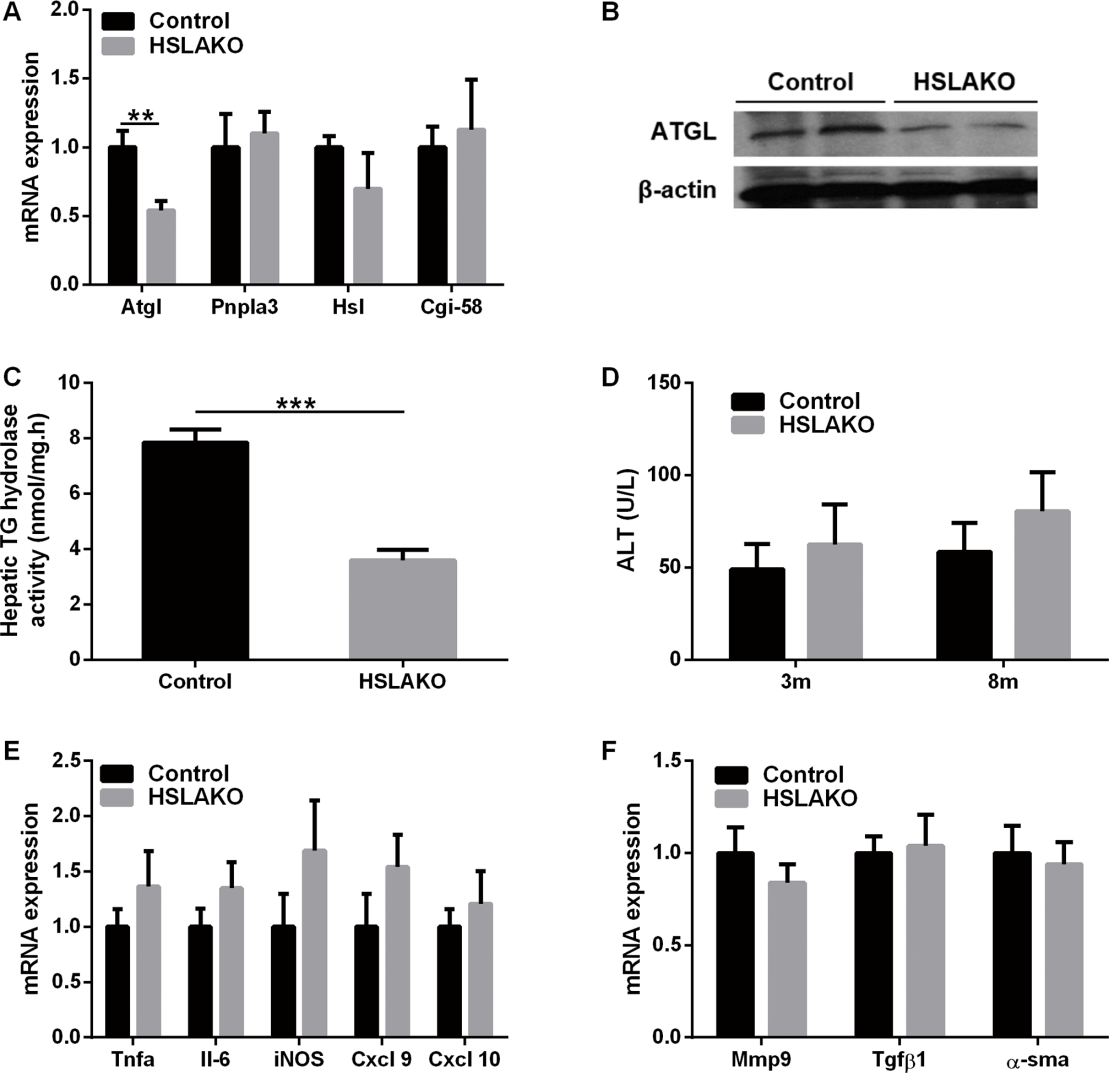
Evaluation of lipolysis, inflammation and fibrosis in HSLAKO livers. A. transcripts related to TG degradation (n = 6). B. Western blot of hepatic ATGL. C. hepatic TG hydrolase activity. D. plasma ALT levels (n = 6). E. inflammation-related transcripts (n = 6). F. fibrosis-related transcripts (n = 6). 8-month-old mice were studied. ATGL, adipose triglyceride lipase; ALT, alanine aminotransferase.

Defective hepatic lipolysis is another potential contributor to steatosis [13]. We therefore measured the mRNA and protein levels of ATGL, the major hepatic TG hydrolase [13]. In liver, HSLAKO mice had lower levels of ATGL mRNA and protein compared to normal controls (Fig 7A–7B), suggesting a lower lipolytic capacity. When hepatic TG hydrolase activity was measured directly with radiolabeled TG as substrate, the activity of HSLAKO liver was 46% that of control mice (Fig 7C). Together, these results suggest a lower capacity for hepatic TG degradation in HSLAKO than in normal control liver.

In summary, lipid metabolism in HSLAKO liver is characterized by low levels of hepatic FA oxidation, of VLDL secretion and of TG hydrolase activity, each of which could contribute to hepatic steatosis.

### The hepatic steatosis of HSLAKO mice is not associated with hepatocyte damage, hepatic inflammation or fibrosis

Plasma levels of ALT (Fig 7D) and liver expression of mRNAs of the pro-inflammatory cytokines TNFα and IL-6 and of the M1 macrophage markers iNOS, Cxcl9 and Cxcl10 were similar in HSLAKO and normal mice (Fig 7E). The levels of three fibrosis-related transcripts, MMP9, TGFβ1 and α-SMA, were also similar in HSLAKO and normal control mice (Fig 7F). Together, these results demonstrate that at 8 months of age, HSLAKO mice develop isolated hepatic steatosis.

## Discussion

**Fig 8:**
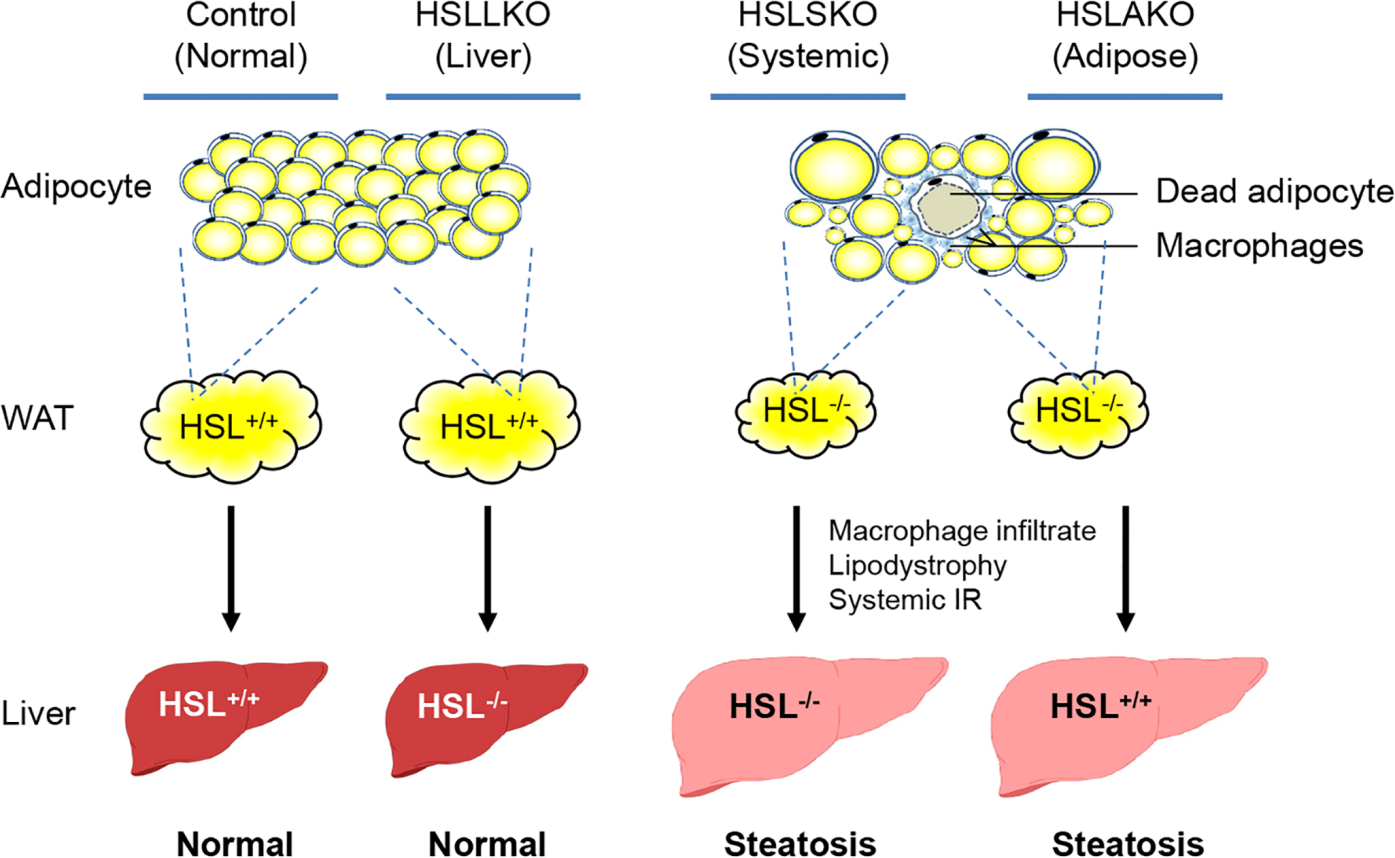
Hepatic steatosis in HSL deficiency is driven by adipose HSL deficiency. Comparison among the four mouse strains studied reveals the mechanism of hepatic steatosis in HSL deficiency: normal controls, systemic HSL-deficient mice (HSLSKO), adipose HSL-deficient mice (HSLAKO), liver HSL-deficient mice (HSLLKO). HSL deficiency in adipose tissue is sufficient to cause hepatic steatosis of a similar degree to systemic HSL deficiency. In contrast, HSL deficiency in liver has no detectable impact on hepatic fat content. WAT: white adipose tissue.

In this study, we showed that age-dependent hepatic steatosis and insulin resistance develop in HSL-deficient mice and that this occurs by an adipose tissue-dependent mechanism (Fig 8). Mice with systemic or adipose HSL deficiency show marked macrophage infiltration in adipose tissue and progressive development of lipodystrophy. In striking contrast, mice with hepatic HSL deficiency had normal liver weight and fat content, excluding a cell-autonomous effect of HSL deficiency on hepatocyte TG content. Together, these results show that adipose HSL deficiency has a major effect on liver TG content.

Adipose tissue influences systemic energy balance. Conditions like exogenous obesity [27, 28] and lipoatrophy [23, 29] reduce the capacity of adipose tissue to take up and to store additional triglycerides, and they are associated with systemic insulin resistance and hepatic steatosis. A small number of mouse models with adipose-specific genetic changes have been reported to develop hepatic steatosis [29–31]. This group includes mice with adipose-specific deficiency of the insulin receptor [29], or of Raptor/mTORC1 [31], both of which develop lipodystrophy, and mice with adipose-specific overexpression of RBP-4 which show adipose tissue inflammation with macrophage infiltration [30]. Congenital and acquired lipodystrophies cause insulin resistance and hepatic steatosis [23, 24]. The insulin receptor, Raptor/mTORC1 and RBP-4 each has direct links to insulin signaling [32–37]. In HSLSKO mice, “crown-like structures” (dead adipocytes surrounded by macrophages) occur [38], as they do in humans with metabolic syndrome [39]. HSLSKO and HSLAKO mice therefore have several features in common with the other models of secondary, adipose-driven hepatic steatosis, including adipose macrophage infiltration and inflammation, age-related progressive lipodystrophy and systemic insulin resistance.

Young HSLAKO mice exhibit improved insulin tolerance despite unchanged liver TG content and body weight. Insulin sensitivity is affected by at least four tissues: skeletal muscle, liver, pancreas, and fat tissue [40]. In young HSLAKO mice, fat tissue is relatively normal (not lipodystrophic yet) compared to the old HSLAKO mice, their skeletal muscle and liver have similar amounts of fat as controls and we detected no evidence of muscle, liver or pancreatic dysfunction. HSLAKO mice show low plasma FFA, likely due to their primary deficiency of adipocyte lipolysis. When other tissues function normally, low plasma FFA levels contribute to improved insulin sensitivity, as previously shown in the ATGL adipose tissue knockout mice [41]. Therefore, the main contributor to improved insulin tolerance in young HSLAKO mice is lower plasma FFA.

Of note, we found that HSLAKO livers showed lower rates of fat disposal by oxidation, VLDL production and lipolysis. In general, there are two potential causes for this. (1) Less availability of fatty acid substrate for these processes is one potential cause. For example, liver specific deficiency of ATGL, the main lipase responsible for hepatic triglyceride degradation, causes TGs accumulation in liver. In these mice, due to lack of FA availability, FA oxidation and VLDL package were low [13]. (2) Impaired subcellular organelle function could be another reason. High TG levels in hepatocytes has been associated with ER stress and/or mitochondrial dysfunction [42], with subsequent lower rates of fat oxidation and VLDL production. In HSLAKO mice, lower plasma FA due to defective lipolysis with reduced availability of fatty acid substrates is one direct cause of lower mitochondrial FA oxidation. Also, when equal among of FAs were given to HSLAKO liver, lower fatty acid oxidation rate were shown in HSLAKO mice than controls (Fig 6D) suggesting an impaired mitochondrial function. Therefore, both substrate availability and dysfunctional subcellular organelle contribute to lower hepatic fatty acid disposal in HSLAKO mice.

Although in adipose tissue, two major lipases ATGL and HSL catalyze sequential steps in lipolysis, their deficiency causes fatty liver in completely different fashions. The secondary hepatic steatosis that occurs in systemic and adipose HSL deficiency contrasts with primary hepatic steatosis in liver-specific ATGL deficient mice as we showed previously [13]. Defining the mechanisms of hepatic steatosis is necessary to individualize treatment. For the hepatic steatosis of HSL-deficient mice, adipose tissue, not liver, should be considered as a main target for prevention and treatment.

The HSL-deficient patients described to date and the 8-month-old HSLAKO mice both develop partial lipodystrophy, with inflammation and low levels of adipogenic and lipogenic markers in adipose tissue. These similarities suggest that the development of hepatic steatosis in HSL-deficient patients may also be mechanistically similar to that of HSL-deficient mice. If so, adipose tissue may be a therapeutic target for preventing and treating hepatic steatosis in patients with HSL deficiency and possibly other forms of secondary hepatic steatosis.

## Materials and methods

### Ethics statement

All experiments were approved by Animal Facility Committee of CHU Sainte-Justine Hospital (protocol 620) according to the guidelines of the Canadian Council on Animal Care (http://www.ccac.ca/en_/).

### Animals

Mice from the previously-described strain of systemic HSL knockout (HSLSKO) mice [17] were bred to a C57BL/6J background for at least 8 generations. Liver-specific HSL knockout mice (HSLLKO) were created by breeding a gene-targeted HSL allele with Lox sites flanking exon 1 of *Lipe* as we previously described [22], with a transgene expressing Cre recombinase from the albumin promoter. Mice with adipose HSL deficiency (HSLAKO mice) were created as we previously described [22]. Controls were sibling littermates with wild type HSL alleles that expressed the albumin-Cre transgenic mice in the controls for HSLLKO mice, and the Fabp4-Cre transgene in the controls for HSLAKO mice. After weaning, mice received Global Rodent Diet (Teklad #2019). All the mice were transferred to a C57BL/6J background for at least eight generations. Male mice were used for all experiments.

### Real time reverse transcriptase PCR and Western blotting

PCR and Western blotting were performed as described [13]. Primers for PCR are listed in S1 Table. Antibodies for Western blotting were: ATGL (#2138, Cell Signaling Technology, Danvers, MA); HSL [43]; and TGH (a gift from Richard Lehner, University of Alberta, Edmonton) [44].

### Plasma metabolite and adipokine measurements

Commercial kits were used to assay plasma fatty acids (FA) (Wako HR Series NEFA-HR, Wako Pure Chemical Industries, Chou-ku, Osaka), TG (12016648 122, Roche Diagnostics, Indianapolis, IN), glucose (ALL-IN-ONE blood glucose monitoring system, ACCU-CHEK Compact Plus, Roche Diagnostics, Indianapolis, IN) and 3-hydroxybutyrate (3-HB) (Precision Xtra blood glucose & ketone monitoring system, Abbott Diabetes Care, Mississauga, Ontario). Hormones were measured with commercially-available kits: insulin (80-INSMSU-E01, Alpco Diagnostics, Salem, NH), leptin (MOB00, R&D Systems, Minneapolis, MN) and adiponectin (MRP300, R&D Systems, Minneapolis, MN).

### Hepatic and skeletal muscle TG analysis

Lipids were extracted from liver and skeletal muscle by the Folch method [45]. Lipid classes were resolved by thin-layer chromatography and TG content was measured as described [13].

### Histology

Tissue fragments were fixed in buffered formalin, then paraffin-embedded for hematoxylin-eosin or Masson trichrome staining. Image J was used for adipocyte diameter measurements. The distribution of cell size was expressed as percentage of total counted adipocytes. A minimum of 6 high power fields (X200) were counted per mouse. Four mice of each genotype were studied.

### Insulin tolerance test (ITT) and glucose tolerance test (GTT)

ITT and GTT were performed as described [13].

### Hepatic FA oxidation

Hepatic FA oxidation was tested as described, using 1-^14^C-palmitic acid (Pekin Elmer) as substrate [13].

### Hepatic secretion of very low density lipoprotein (VLDL)

Hepatic VLDL secretion was measured as described [13].

### Hepatic TG hydrolase activity

This was assayed *in vitro* using Triolein [42] (Perkin Elmer) as substrate, as described [13].

### Statistical analysis

Values are presented as means ± SEM. Groups were compared using the unpaired two-tailed Student’s t-test.

## Supporting information

S1 Fig Undetectable HSL protein and low *Lipe* mRNA in liver of HSLLKO mice. Livers from 5-hour-fasted 8-month-old mice were used. A. Western blot of HSL. B. mRNA levels of *Lipe*, measured by real-time PCR.
(TIF)

S1 Table Primers used for real-time PCR. This table describes the primers used for evaluation of mRNA expression in liver and adipose tissue.
(TIF)

S1 File Underlying data for the graphs and bar charts. This file describes the original underlying data for the graphs and bar charts shown in figures in spreadsheet form (N = 6).
(XLSX)
