# Supplementary figures and images for: Adipose tissue deficiency of hormone-sensitive lipase causes fatty liver in mice
Source: PLoS Genet. 2017 Dec 12;13(12):e1007110. doi: 10.1371/journal.pgen.1007110 (PMC5741266; doi:10.1371/journal.pgen.1007110)

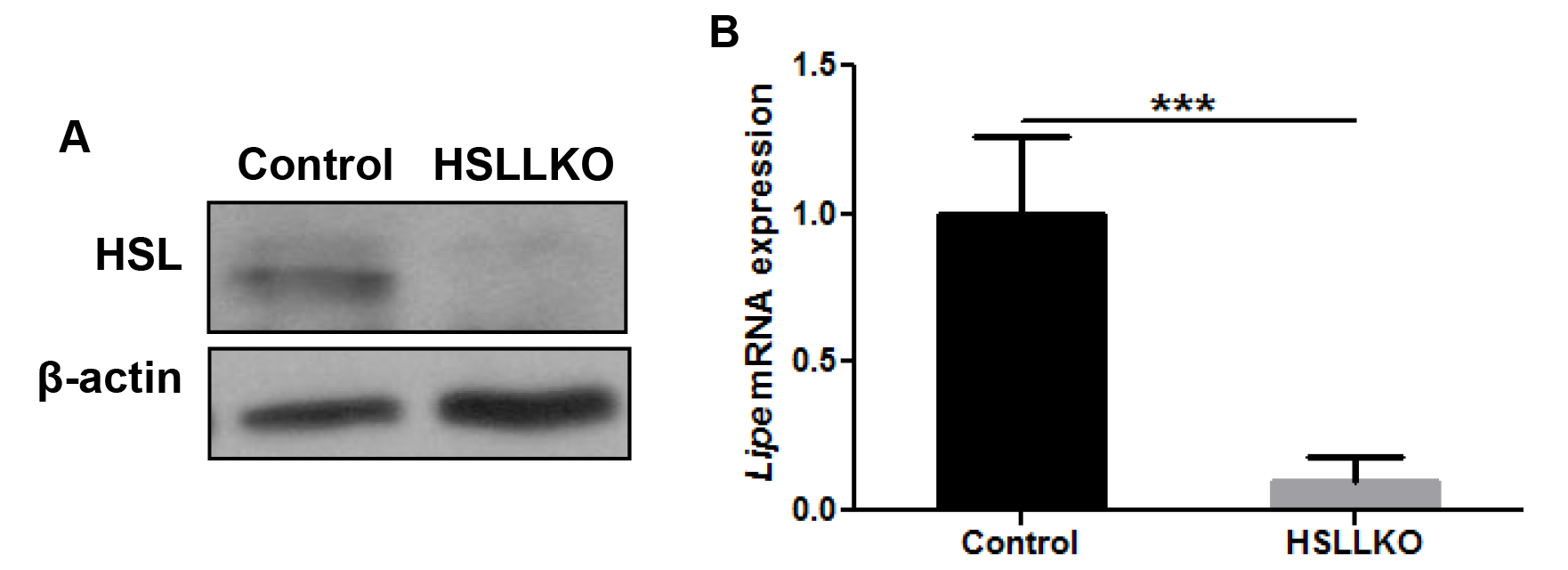

Supplement: S1 Fig — Livers from 5-hour-fasted 8-month-old mice were used. A. Western blot of HSL. B. mRNA levels of Lipe, measured by real-time PCR. (TIF) [file pgen.1007110.s001.tif]

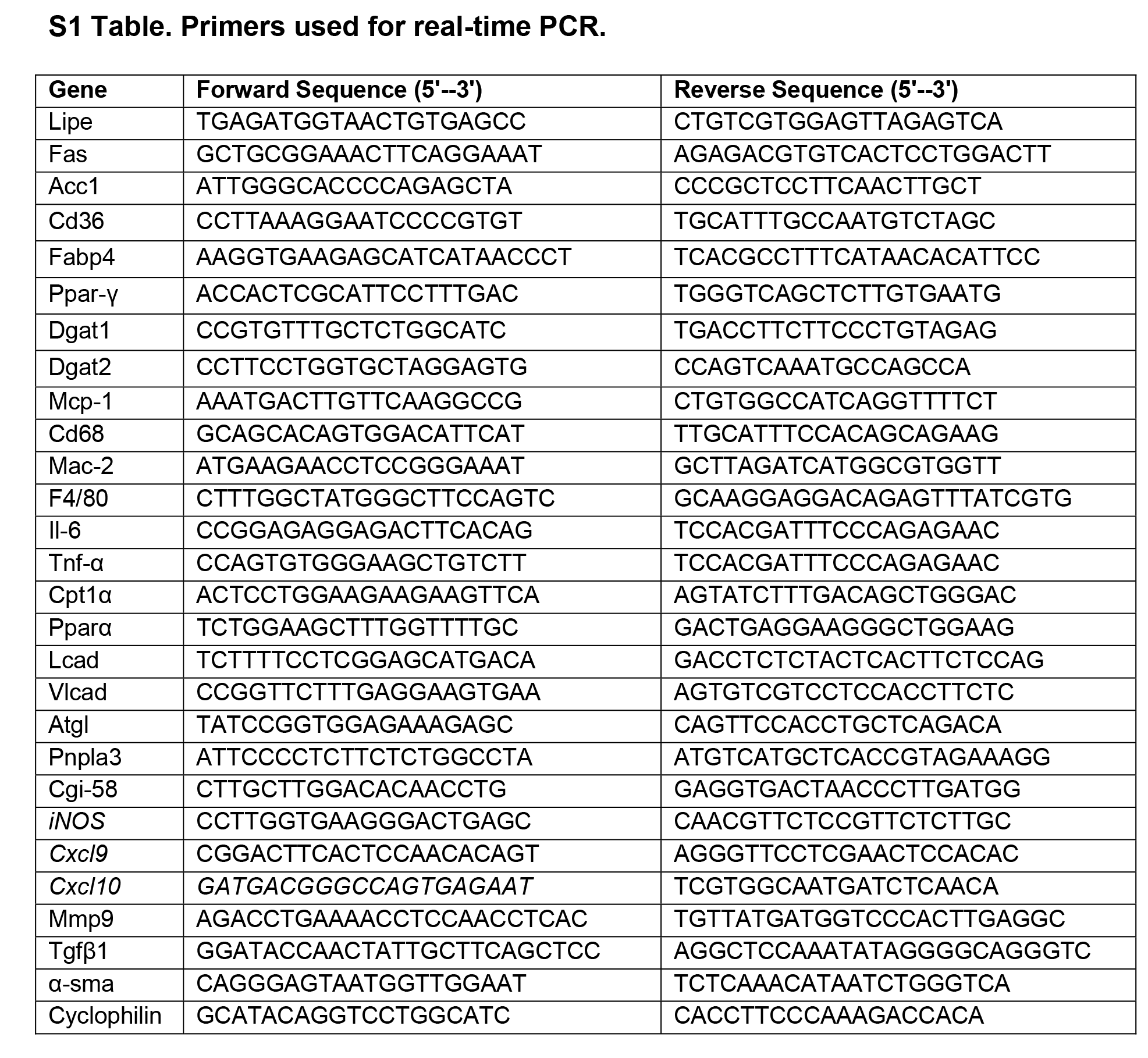

Supplement: S1 Table — This table describes the primers used for evaluation of mRNA expression in liver and adipose tissue. (TIF) [file pgen.1007110.s002.tif]
